# Comparison of birth weights of neonates of mothers receiving vs. not receiving zinc supplement at pregnancy

**DOI:** 10.1186/s12884-021-03598-8

**Published:** 2021-03-06

**Authors:** Hassan Boskabadi, Gholamali Maamouri, Javad Akhondian, Farah Ashrafzadeh, Abbas Boskabadi, Raheleh Faramarzi, Elahe Heidar, Nafiseh Pourbadakhshan, Seyed Reza Habibzadeh Shojaei, Maryam Zakerihamidi, Atiyeh Mohammadzadeh Vatanchi, Mohammad Sokhtanloo, Naghmeh Razaghi, Farnaz Kalani, Hosein Ataei, Azadeh Darabi, Mojgan Sadat Mousavi, Saeideh Hakimiakhangan, Fathemeh Bagheri

**Affiliations:** 1 Department of Pediatrics, Faculty of Medicine, Mashhad University o Medical Sciences, Mashhad, Iran; 2 Department of Orthopedic Surgeon, Faculty of Medicine, Faculty of Medicine, Mashhad University of Medical Sciences, Mashhad, Iran; 3 Department of Midwifery, School of Medical Sciences, Tonekabon Branch, Islamic Azad University, Tonekabon, Iran; 4 Department of Gynecology, Faculty of Medicine, Mashhad University of Medical Sciences, Mashhad, Iran; 5 Department of Biochemistry, Faculty of Medicine, Mashhad University of Medical Sciences, Mashhad, Iran; 6 Nursing and Midwifery Care Research Center, Mashhad University of Medical Sciences, Mashhad, Iran; 7 Department of Pediatrics, Fellowship of neonatology, Mashhad University of Medical Sciences, Mashhad, Iran; 8 Neonatal Intensive Care, Faculty member of Mashhad University of Medical Sciences, Mashhad, Iran; 9 Neonatal Intensive Care, Faculty member of Azad University, Mashhad, Iran

**Keywords:** Zinc, Pregnancy, Birth weight, Neonate, Supplements

## Abstract

**Background:**

Zinc is an essential element for normal embryogenesis and embryonic and neonatal development. Therefore, we compared the birth weights of neonates born to mothers who consumed zinc supplement during pregnancy with that of neonates born to mothers who did not.

**Methods:**

In a cross-sectional study, we divided 200 pregnant mothers into two groups: case group (mothers receiving zinc supplement during pregnancy) and control group (mothers not receiving zinc supplement during pregnancy) Then, the neonate’s cord zinc level and mother’s serum level were measured and neonate’s growth charts (weight, height and head circumference)were completed.

**Results:**

In this study, both groups of mothers were observed to have zinc deficiency; 35% of the mothers who consumed zinc supplements and 81% of the mothers who did not consume zinc supplements (*P* < 0.001). Based on the results, maternal serum of zinc (*P* < 0.001), neonatal birth weight (*P* = 0.008), maternal age (*P* < 0.001) and parity (*P* < 0.01) in zinc-supplemented group were higher. Neonatal birth weight was associated moderately with mother’s zinc serum levels and poorly with neonatal serum zinc levels.

**Conclusion:**

Zinc consumption during pregnancy increases serum zinc level of mother and neonatal weight. Neonatal weight has a higher correlation to maternal serum zinc level.

## Background

Birth weight is one of the major determinants of the future development of a child’s growth, both physically and neurologically, a valid indicator of intrauterine growth, and an important indicator of neonate health [1]. It is also considered a factor in measuring pregnancy outcome [2]. This index is related to maternal health, quality of prenatal care, socio-economic factors [3] and maternal nutrition quality [4]. Low birth weight (weight below 2500 g) leads to higher mortality, disability, and morbidity in childhood [5].

Pregnancy is associated with an increase in the need for micronutrients, including zinc, and a decrease in these can affect the prognosis of pregnancy [6, 7]. Zinc plays an important role during embryogenesis and embryo development. Therefore, the need for zinc during pregnancy is greater. Increased dietary zinc and zinc uptake, reduced zinc loss, and altered zinc production in the body can lead to increased body zinc requirement in pregnancy [8]. Walt et al. stated that providing nutritional supplements during pregnancy is essential for fetal growth, and that deficiencies in vitamins and minerals, especially folic acid, vitamin B12, calcium, iron and zinc, are associated with adverse pregnancy outcomes and has negative side effects on fetal health, growth and development [9]. Zinc deficiency has also been associated with neonatal jaundice [10]. However, Zinc consumption in newborns has not been shown to decrease the incidence and severity of jaundice [11].

During pregnancy, due to a higher number of red blood cells, embryonic growth and placenta formation, maternal need for supplements is increased. If supplements are inadequate, contrastingly, increased maternal plasma volume and physiological anemia in pregnancy can lead to an inadequate oxygen supply and trace elements for the embryo; and this, in turn, may interfere with fetal growth. Given the high cost of caring for low birthweight neonates and their consequent health issues, postpartum care for these neonates has been the focus of researchers for decades with the intent of improving their prenatal care conditions [12]. The present study objective is to compare the birth weights of neonates whose mothers consumed zinc supplement in pregnancy with the weight of those whose mothers did not.

## Methods

### Study design and population

This cross-sectional study was carried out on neonates born from 2016 to 2019 to 109 (66.1%) mothers who did not use zinc supplement during pregnancy (control group) and 56 (33.9%) mothers who used zinc supplement during pregnancy (case group). After obtaining the approval of ethics committee and the code 950082, from Mashhad University of Medical Sciences)IR.MUMS.fm.REC.1395.238(, and obtaining informed written consent from pregnant mothers. Mothers with a gestational age of more than 37 weeks who presented labor pains were assessed for zinc supplementation. Mothers with a history of at least 3 months of zinc intake, at least 25 mg daily, were included in the study and mothers with a history of not using zinc supplements were considered as the control group. According to Saaka et al. study, zinc-supplemented mothers had a 60% increase in serum zinc levels, while the non-zinc supplemented mothers had a 30% increase in serum zinc levels. In our study, we used the formula of comparing two ratios related to an attribute from two communities and α = 0.05 and β = 0.2 = sample size. A minimum of 56 people was included in each group [13].

### Inclusion and exclusion criteria

Inclusion criteria included live neonates over 37 weeks of gestation born to apparently healthy mothers with single pregnancy. Exclusion criteria included the following; mothers who did not use zinc regularly (no more than 2 days a week), mothers with specific underlying disease (preeclampsia, gastrointestinal diseases, hypertension, infections, malignancy, bone and endocrine disorders, history of anticonvulsant and neurologic medication use),mothers with multiple newborns, or very ill neonates who died within the first 24 h, neonates with severe asphyxia, cases with incomplete information, and probability of infection in the sampling tube.

### Anthropometric measures

Data was collected through checklists and face-to-face interviews with mothers, review of maternal and neonatal medical records, and a neonatal examination by a neonatologist. For neonates in our study, information including sex, gestational age, birth weight, head circumference and height, as well as maternal information including maternal age, parity, type of delivery and zinc supplement consumption was gathered and recorded. All neonates were weighed at birth by a midwife using the Misaki Balance made in Japan with 50 g accuracy; head circumference and height were also measured. Neonates of less than 2500 g are defined as low birth weight (LBW).

### Biochemical measures

At birth, the neonate’s umbilical blood cord is usually discarded and about 2 cc of blood is collected from mother simultaneously. The serum is separated and sent to a laboratory in dark or coated tubes to measure the zinc content. Before sampling, the serum tubes were thoroughly rinsed with acid and deionized water to remove trace elements and after 10 min the serum was centrifuged (1000 rpm) in polyethylene tubes. Serums were obtained from both groups and kept in a freezer at − 70 degrees Celsius. After unfreezing, all serum samples were diluted by 1% nitric acid. Zinc level was measured by atomic absorption spectrophotometer (Perkin-Elmer 2380; Perkinelmer, Wellesly, MA, USA) through preparing suitable calibration graphics of wavelengths for each parameter. Mothers and neonates with zinc levels below 60 μg / dl were considered zinc deficient.

### Statistical methods

After collecting and recording the data in SPSS Software (version 23 SPSS Inc., Chicago, Illinois, USA), we examined it using tables, graphs, and statistical indicators. First, the distribution status of the variables was investigated using the Kolmogorov-Smirnov test. For comparing the mean of quantitative variables between the groups, independent t-test or nonparametric equivalent, Mann-Whitney test, was used. For comparison of the qualitative variables between the studied groups, chi-square test was used. Spearman and Pearson correlations were used to determine the correlation serum zinc level of mothers & neonates, and birth weight *P*-value of less than 0.05 was regarded as statistically significant.

## Results

Of the 200 mothers studied, 16 infants with improper zinc supplement use, 8 neonates requiring resuscitation in the operating room, 6 neonates requiring hospitalization in the first week, and 5 samples due to probability of tube contamination were excluded. In this study, 56 (33.9%) of pregnant mothers used zinc supplement (case group) and 109 (66.1%) pregnant mothers did not (control group). One hundred and two neonates (61.8%) were born by cesarean section and 63 neonates (38.2%) by vaginal delivery. Seventy-six neonates (51.4%) were male and seventy-two neonates (48.6%) were female. Serum zinc levels in 60% of mothers and 33% of neonates were less than 60 μg / dl. Eighty-one percent of mothers who had not consumed zinc supplement during pregnancy had zinc deficiency, while 35% of mothers consuming zinc supplement had deficiency during pregnancy (*P* < 0.001).Zinc deficiency was seen in 31 % of neonates of mothers who had not consumed zinc supplement during pregnancy, and in 28% of neonates of mothers who had consumed zinc supplement during pregnancy (*P* = 0.221).

Both mothers of neonates weighing less than 2500 g and mothers of neonates weighing more than 2500 g; 70% and 28% respectively, were found to have deficiency of zinc (*P* < 0.001). Thirty-seven percent of neonates weighing less than 2500 g and 33 % of neonates weighing more than 2500 g had zinc deficiency (*P* = 0.427).

In this study, there were significant differences in maternal serum zinc level (*P* < 0.001), birth weight (*P* = 0.008), maternal age (*P* < 0.001) and parity (*P* = 0.001) between the two groups of neonates, namely, those born to mothers with zinc supplement intake and those born to mothers without it. These results confirm that the values ​​of these variables were higher in neonates with zinc-supplemented mothers. There was no significant difference between the two groups of neonates in neonatal serum zinc level (*P* = 0.626), head circumference (*P* = 0.193) and height (*P* = 0.313) (Table 1).

**Table 1:** Comparison of the maternal and neonate background characteristics between neonates with mothers without zinc supplement and neonates with mothers with zinc supplement

Variable	Neonates with maternal zinc supplement 56 Neonates(33/9%) mean ± SD	Neonate without maternal zinc supplement 109 Neonates(66/1%) mean ± SD	***P***-Value (T-Test)
serum zinc level of Mother (μg/dl)	68/36 ± 17/17	50/82 ± 17/42	0/000
Neonatal serum zinc level (μg/dl)	64/95 ± 21/62	62/61 ± 17/65	0/626
Birth weight (gr)	3096/50 ± 577/55	2828/28 ± 617/84	0/008
Head circumference (cm)	34/26 ± 2/62	33/55 ± 2/09	0/193
Height (cm)	49/04 ± 3/82	48/18 ± 3/41	0/313
Maternal age (year)	35/36 ± 6/01	29/62 ± 6/82	0/000
Parity	3/00 ± 1/53	2/08 ± 1/17	0/001

**Fig. 1:**
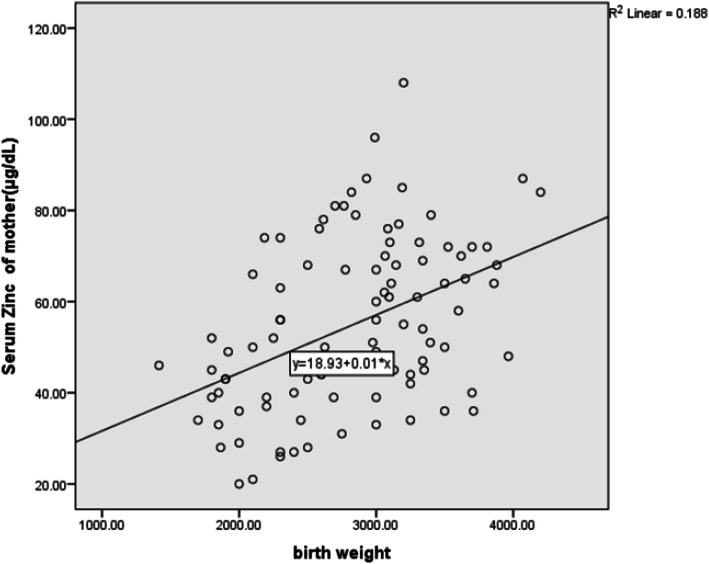
Relationship between birth weight and maternal serum zinc

**Fig. 2:**
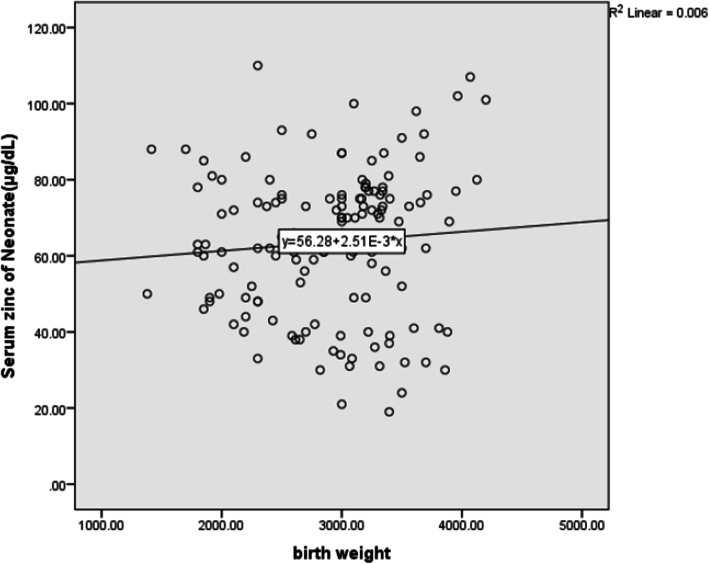
Relationship between birth weight and neonatal serum level

The difference between the two groups in terms of sex (*P* = 0.398) and gestational age (*P* = 0.474) was not statistically significant. The results show that birth weight is moderately correlated with maternal serum zinc and poorly correlated with neonatal serum levels (Table 2, Fig. 1, 2).

**Table 2:** comparison of relationship between birth weight and maternal and neonatal serum level

Variant	Paired Samples Correlations	Paired Samples Correlations (Pearson) Sig.	Mean ± Standard deviation
Birth weight (gr)	0/311	0/002	55/577 ± 50/3096
Maternal zinc level (μg/dl)			17/17 ± 36/68
Birth weight (gr)	0/074	0/378	55/577 ± 50/3096
Neonatal zinc level (μg/dl)			62/21 ± 95/62

## Discussion

Sixty percent of mothers and 33% of neonates had zinc levels lower than 60 μg / dl. In this study, one-third of pregnant women used zinc supplements during pregnancy. Although severe zinc deficiency is relatively rare, mild to moderate zinc deficiency is very common. It is estimated that about 59.9% of pregnant women and 38.4% of children have zinc deficiency [14]. Recent estimates have shown that about half a million deaths of mothers and children due to zinc deficiency occur annually worldwide, which raises widespread concerns about insufficient zinc intake by pregnant women in developing countries [15]. It is also estimated that approximately 50% of women of reproductive age suffer from zinc deficiency. Zinc deficiency in mother’s serum reduces zinc transfer to the fetus and increases the risk of developmental defects. It plays important structural and enzymatic roles in many proteins. Part of the teratogenic effects of zinc deficiency may be due to alterations in the activity of proteins in the fetus [16].

Seventy percent of mothers of LBW infants had zinc levels less than 60 μg / dl while 28% of mothers of neonates weighing more than 2500 g had zinc levels less than 60 μg / dl (*P* < 0.001). According to the results of one study, serum zinc levels were significantly lower in LBW neonates than in normal neonates. Zinc levels were also lower in mothers of LBW neonates as compared to mothers of neonates with optimum birth weight. Low maternal zinc levels are associated with low zinc levels in LBW neonates [17]. Gupta found that zinc levels in the serum of Small for Gestational Age (SGA) neonates is lower than neonates with appropriate weight. It has also been shown that zinc deficiency is more common in preterm SGA newborns. SGA neonates have significant zinc deficiency as compared to AGA neonates. This zinc deficiency is even more pronounced in SGA newborns that are born preterm. Therefore, use of zinc supplementation during pregnancy especially for preterm and SGA babies can promote positive outcomes of maternal and child health [18].

According to the results of this study, 81% of mothers who had not consumed zinc supplement during pregnancy had zinc levels lower than 60 μg / dl while 35% of mothers having consumed zinc had zinc levels lower than 60 μg / dl during pregnancy (*P* < 0.001). Gholizadeh showed that high concentration of zinc in mother’s serum was directly associated with dietary intake zinc and supplement zinc. Hanachi indicated that the zinc deficiency in the latter third of pregnancy may suggest insufficient maternal nutrition [19]. There are no valid tools for measuring nutrition zinc adequacy. This evaluation is difficult because of the fact that all digested zinc is not consumed by the organism and its bioavailability may be affected by intestinal absorption or blood circulation. Intestinal absorption may be reduced by dietary antagonistic factors such as phytates, oxalates, tannins, and polyphenols, while circulating zinc may compete with copper and iron, depending on the amount of these elements in the bloodstream [20]. Lack of response to zinc supplement intake (lack of proper serum increase) may be due to lack of gestational age control or serum albumin concentration or low sample size. Despite these limitations, serum zinc concentration is currently used as a biochemical indicator of zinc status during pregnancy and is an indicator to evaluate the effect of zinc supplement in pregnant women [21].

According to the results of our study, 31% of neonates of mothers who did not use zinc during pregnancy had a zinc level less than 60 μg / dl while 28% of neonates of mothers who consumed zinc during pregnancy had a zinc level less than 60 μg / dl (*P* = 0.221). Placental transfer and release into the baby’s blood is an active process. The concentration of zinc in the umbilical cord serum was 14 μmol /L and the concentration of zinc in the mother serum was 6 to 15.6 μmol /L [22].

Therefore, zinc deficiency in neonates occurs only in cases of severe zinc deficiency in the mother and due to the function of placental transfer of zinc to the fetus, neonatal zinc levels are usually higher than the mother’s zinc levels [8]. Possibly due to the transfer of the mother’s zinc stores to neonates, zinc levels did not decrease among neonates of mothers not consuming zinc, similar to the group of neonates whose mothers did consume zinc.

In this study, serum zinc levels of mothers receiving zinc supplement were higher (*P* < 0.001). Zinc levels during pregnancy are affected by dietary zinc intake. Physiological changes in zinc metabolism in pregnancy include changes in the tissue distribution of zinc in the body, increased zinc uptake, decreased endogenic zinc loss, and changes in zinc production [8].

In one study, a higher zinc supplement of 25 mg/day was given to mothers. Zinc concentration in maternal plasma was evaluated. It declined during pregnancy irrespective of using zinc supplements. This supplement increased zinc levels in maternal erythrocyte as compared to women not using zinc supplements [22]. In another study, the neonatal zinc level was higher than their mothers. It seems placental transfer and release into the baby’s blood is an active process as a result of high zinc requirement in the fetus. On the other hand, it could be caused by increased absorption in the fetus and reduction of maternal zinc during pregnancy due to physiologic dilution of maternal blood volumes [6].Therefore, it seems reasonable that zinc consumption in the mother would lead to an increase in maternal serum levels.

Birth weight of neonates consuming zinc supplement was higher (*P* = 0.008). According to the results of this study, the birth weight of neonates had a moderate correlation with maternal serum zinc level and a weak correlation with neonatal serum zinc level. Low concentrations of zinc in the mother’s serum reduce placental transfer to the fetus. Therefore, the use of multivitamin supplements containing zinc is recommended to all pregnant women in developing countries since zinc supplement significantly improves weight gain and growth in children. However, other factors (nutritional and environmental) can also contribute to child growth [23].

Based on the results of one study, maternal zinc supply in mid-pregnancy from animal sources was positively correlated with neonatal birth weight and height [24]. Results of another study showed that prenatal iron and zinc supplement may increase neonatal birth weight in anemic and iron deficient mothers, but in mothers with high iron stores early in pregnancy, these supplements may not affect neonatal birth weight [13]. A researcher in Tanzania found that mothers who had low levels of zinc at birth had a two-and-a-half-fold increase in the likelihood of having babies weighing less than 2000 g compared to mothers with normal zinc levels [25]. In Kadem’s study, zinc supplement intake by pregnant women had a positive effect on birth weight and head circumference [26]. Maamouri et al. showed that there was a relationship between birth weight below 2500 g and maternal zinc level and that the risk of having babies weighing less than 2500 g is 3.8 times higher in mothers with zinc levels less than 6.4 mol/l [6]. As our study shows, consuming zinc in the mother significantly increases the birth weight and may reduce the birth of LBW neonates.

## Conclusion

The results of the present study showed that one third of pregnant women use zinc supplement. Two-thirds of mothers of LBW neonates and four-fifths of mothers who had not consumed zinc supplement during pregnancy had zinc levels lower than 60 μg / dl, while one third of mothers consuming zinc supplement during pregnancy had zinc levels less than 60 μg / dl. Although zinc consumption by mothers did not significantly increase serum Zinc level of newborn, it did increase the neonates’ weight. Furthermore, birth weight had stronger correlation with mothers’ Zinc levels than with newborns’ Zinc levels. Concerning the importance of birth weight in neonates’ mortality and complications, Zinc consumption by mothers might improve the weight of these newborns and supplementation of zinc (as well as other micronutrients) should be considered essential..

## Data Availability

If needed, patient data will share. Research data are available within the manuscript and/or additional supporting files. Identifying/confidential patient data should not be shared.

## Abbreviations

LBW: Low birth weight
SGA: Small for Gestational Age
